# Potential Role of Vegetarianism on Nutritional and Cardiovascular Status in Taiwanese Dialysis Patients: A Case-Control Study

**DOI:** 10.1371/journal.pone.0156297

**Published:** 2016-06-13

**Authors:** Shih-Hsiang Ou, Mei-Yin Chen, Chien-Wei Huang, Nai-Ching Chen, Chien-Hsing Wu, Chih-Yang Hsu, Kang-Ju Chou, Po-Tsang Lee, Hua-Chang Fang, Chien-Liang Chen

**Affiliations:** 1 Division of Nephrology, Kaohsiung Veterans General Hospital, Kaohsiung, Taiwan; 2 Division of Nutrition, Kaohsiung Veterans General Hospital, Kaohsiung, Taiwan; 3 Department of Medicine, National Yang-Ming University School of Medicine, Taipei, Taiwan; 4 Kaohsiung Chang Gung Memorial Hospital, Chang Gung University, College of Medicine, Kaohsiung, Taiwan; Postgraduate Medical Institute, INDIA

## Abstract

**Background & Objectives:**

Cardiovascular disease remains the most common cause of death for patients on chronic dialysis. End stage renal disease patients undergoing dialysis imposed to reduce phosphorus intake, which likely contributes to development of vegetarian diet behaviors. Vegetarian diets are often lower in protein content, in contradiction to the recommendation that a high protein diet is followed by patients undergoing dialysis. The purpose of the study was to investigate the effects of a vegetarian diet on the nutritional and cardiovascular status of dialysis patients.

**Design, Setting, Participants, Measurements:**

A study of 21 vegetarian dialysis patients and 42 age- and sex-matched non-vegetarian dialysis patients selected as controls was conducted in the Kaohsiung Veterans General Hospital. Brachial-ankle pulse wave velocity and biochemistry data including total homocysteine levels, serum lipid profiles, high-sensitivity C-reactive protein, vitamin D levels, albumin, and normalized protein catabolic rate were measured.

**Results:**

Compared with the non-vegetarian control group, vegetarian subjects had lower body weight, body mass index, serum phosphate, blood urea nitrogen, serum creatinine, vitamin D, uric acid, albumin, and normalized protein catabolic rate (*p* < 0.05). The vegetarian group showed higher brachial-ankle pulse wave velocity than the non-vegetarian group (1926.95 ± 456.45 and 1684.82 ± 309.55 cm/sec, respectively, *p* < 0.05). After adjustment for age, albumin, pre-dialysis systolic blood pressure, and duration of dialysis, vegetarian diet remained an independent risk factor for brachial-ankle pulse wave velocity.

**Conclusions:**

The present study revealed that patients on dialysis who follow vegetarian diets may experience subclinical protein malnutrition and vitamin D deficiency that could offset the beneficial cardiovascular effects of vegetarianism.

## Introduction

Cardiovascular disease (CVD) is the leading cause of morbidity and mortality among patients with end-stage renal disease (ESRD) who are on chronic dialysis [1]. According to the United States Renal Data System, CVD accounts for approximately 40% of mortality among patients on dialysis and is the principal cause of hospitalization [2]. Both traditional and non-traditional risk factors have been implicated in the development of CVD in chronic dialysis patients. Traditional risk factors are those used to predict coronary heart disease outcomes in the general population and include hypertension, smoking, hyperlipidemia, hyperglycemia, and obesity [3]. Non-traditional risk factors are uremia-related factors that increase in prevalence as kidney function declines and contribute to the excess risk of CVD observed in patients with chronic kidney disease (i.e., anemia, abnormal calcium/phosphorus metabolism, hyperhomocysteinemia, extracellular fluid volume overload, oxidative stress, malnutrition, and inflammation) [4]. Recently, increased cardiovascular morbidity and mortality have been correlated with various estimates of elevated left ventricle afterload, such as arterial wall stiffness, in patients on chronic dialysis [5].

Arterial stiffness results from fibro-elastic intima thickening, increased collagen accumulation, and fragmentation of elastic lamellae with secondary fibrosis and calcification of the media. Arterial stiffness has been measured as aortic stiffness using a tonometric sensor on the carotid and femoral arteries [6–8]. Carotid femoral pulse wave velocity (cfPWV) is measured in order to reflect aortic pulse wave velocity. However, this technique is limited by low reproducibility due to its technical difficulty. Brachial-ankle pulse wave velocity (baPWV) is automatically measured by applying blood pressure cuffs over the arms and ankles; this is simple and reproducible compared with cfPWV measurement. Aortic stiffness (cfPWV) and baPWV are correlated [6], and the power of baPWV measurements for predicting the presence of CVD is comparable to that of aortic pulse wave velocity in the general population. Increased arterial stiffness, which can be examined by baPWV, is a powerful and independent predictor of all-cause and cardiovascular mortality in the general population and ESRD patients [7–11].

The number of vegetarians is increasing in many developed countries and different populations, including in people on chronic dialysis. Various dietary restrictions are imposed on dialysis patients in order to control hyperphosphatemia, hyperkalemia, and calcium balance. Dietitians may recommend the reduction of excessive reliance on high phosphorus-to-protein ratio animal sources (i.e., fish, meat, and chicken) and avoidance of milk and milk products (i.e., yogurt and cheese) because these foods have higher phosphorus content. The dietary restrictions that are imposed to reduce phosphorus intake likely contribute to increasing vegetarianism among patients on dialysis.

Vegetarian diets have long been considered beneficial for health. Several investigators have shown that vegetarian diets have a positive impact in altering serum lipids, reducing blood pressure, improving glycemia control and insulin sensitivity, reducing weight, and lowering mortality [12, 13]. However, other studies have found no beneficial effects and indicated that vegetarian diets were associated with the development of subclinical protein malnutrition and vitamin deficiency [14, 15]. The National Kidney Foundation^™^ offers guidelines through the Kidney Disease Outcomes Quality Initiative, recommending an intake of 1.2 g of protein/kg of body weight for people on dialysis, with at least 50% of the dietary protein being of high biological value [16]. The challenge is to ensure that dialysis patients have adequate intake of complete proteins containing all nine essential amino acids; these are mainly found in animal products. Vegetarian diets are often lower in protein, in contrast to the high protein diet recommended for dialysis patients. The present case-control study was designed to investigate the effects of vegetarian diets on nutritional and cardiovascular status, including arterial stiffness, in chronic dialysis patients.

## Subjects and Methods

### Study protocol and participants

An age- and sex-matched case-control study was conducted in the dialysis unit of Kaohsiung Veterans General Hospital in south Taiwan between January 1 and December 31, 2014. The subjects were recruited for an investigation of the effect of Taiwanese vegetarian diets on arterial stiffness and cardiovascular risk profiles in chronic dialysis patients. The study had approved by Kaohsiung Veterans General Hospital Institutional Review Board and each subject provided informed written consent prior to participation. The inclusion criteria were patients aged ≥18 years old, who had received dialysis therapy (including hemodialysis and peritoneal dialysis) for >6 months and remained in a stable condition, and followed vegetarian diet habits. The vegetarian subjects included strict vegetarians and ovo-lactovegetarians, all of whom had consumed a vegetarian diet for ≥18 months prior to starting the study, and did not consume any meat or fish (those who consumed small amounts of milk and egg products were included in the vegetarian group). The patients were denied nutritional vitamin D supplementation during the study, which was confirmed by medical charts and interviews. The exclusion criteria were presence of diabetes mellitus, active malignancy, active inflammation, atrial fibrillation, high-degree atrioventricular block, peripheral arterial occlusion disease (PAOD), or waveform images inadequate for the calculation of baPWV. An ankle-brachial blood pressure index <0.9 was accepted to be a reliable marker for PAOD. In patients who developed acute coronary syndrome, congestive heart failure, peritonitis, exit site infections, other infective complications, or any other complications that required hospitalization, all of the above assessments were deferred for at least 3 months after complete resolution of the complication.

For each case, two non-vegetarian controls, matched with the case by age (within ±5 years) and sex, were randomly selected from hospital lists of patients on chronic dialysis. All control group patients conformed to the study inclusion and exclusion criteria.

### Data collection

Upon entering the study, all patients underwent baPWV measurements. All blood biochemical analyses were conducted on samples obtained before the hemodynamic study. These included serum blood urea nitrogen (BUN), serum creatinine, hematocrit level, albumin, high sensitivity C-reactive protein (Hs-CRP), intact parathyroid hormone (iPTH), uric acid, vitamin B12, homocysteine, normalized protein catabolic rate (nPCR), 25-hydroxyvitamin D (25(OH)D) levels, and lipid profiles. In addition, personal dietary behavior was assessed through interviews conducted by a qualified nutritionist. All participants were asked to keep 24-hour dietary records and were given detailed instructions on how to keep dietary records using household measures. Completed records were checked and analyzed by a qualified nutritionist.

### Biochemical analysis

Fasting blood samples were collected for measuring serum 25(OH) D, albumin, iPTH, alkaline phosphate, plasma homocysteine levels, and lipid profiles. Serum 25(OH)D was measured using ELISA (Immunodiagnostic Systems Inc., Fountain Hills, AZ, USA) that detected both vitamins D2 and D3, with a detection limit of 5.0 nmol/L. Intact PTH was measured using an iPTH immunoradiometric assay (IRMA; Scantibodies Laboratory, Inc., Santee, CA, USA) with a normal range of 8–76 pg/mL. Serum cholesterol was determined with an enzymatic photometric assay using a Hitachi 7600 analyzer (Roche, Hitachi system, Tokyo, Japan). Serum high-density lipoprotein (HDL) and low-density lipoprotein (LDL) levels were determined using homogeneous enzymatic colorimetric assays (Sekisui, Osaka, Japan); the coefficient of variation (CV) was 2.08% and 2.61%, respectively. Serum triglycerides were assessed with an enzymatic photometric assay (CHO-HMMPS method) (WAKO, Osaka, Japan), with a CV of 1.98%. Serum homocysteine and vitamin B12 levels were determined using the chemiluminescent microparticle immunoassay method on an Architect i1000 analyzer (Abbott, MA, USA).

### Assessment of arterial stiffness by baPWV measurement

BaPWV values were assessed using a VP-1000 vascular profiler (Nippon Colin Ltd., Komaki City, Japan) that allowed online pulse wave recording and automatic calculation of PWV. Briefly, baPWV was calculated from the equation: (D1 − D2)/T, where D1 was the distance between the heart and ankle, D2 was the distance between the heart and brachium, and T was the transit time between the right brachial arterial wave and the right tibial arterial wave. Distances between sampling points were automatically calculated from the patient’s height and divided by the time interval for the waveform from each measuring point. Two measurements were performed in each arm and average values were used for the analysis. BaPWV was used as a marker of arterial stiffness on the basis of its ease of measurement, reproducibility, and validity in previous studies [6–8]. All the baPWV measurements were performed by the same experienced operator.

### Statistical analysis

Continuous variables were expressed as mean ± standard deviation. Comparisons between the two groups were performed using the student’s t test or χ^2^ test as appropriate. Non-parametric data were compared using the Mann-Whitney U test, because cardiovascular risk factors influence aortic stiffness. Significant factors determining PWV values were also evaluated, using multiple linear regression models to estimate the effects of vegetarianism after controlling for major CVD risk factors. Statistical significance was set at a two-tailed *p* value< 0.05. All statistical analyses were performed using SPSS statistical software 17.0 for Windows (SPSS, Chicago Davis, IL, USA).

## Results

A total of 63 subjects (21 vegetarians and 42 sex- and age-matched non-vegetarians) were enrolled in this study. According to their clinical characteristics and biochemical parameters, 12 male and 51 females were included, with a mean age of 56.27 ± 12.28 years. Causes of ESRD in the study group included chronic glomerulonephritis (n = 22; 34.9%), chronic interstitial nephritis (n = 32; 50.8%), autosomal dominant polycystic kidney disease (n = 2; 3.2%), IgA nephropathy (n = 5; 7.9%), and lupus nephritis (n = 2; 3.2%). The median and average durations of dialysis were 115 months and 125 ± 70 months, respectively. The vegetarian group contained 18 lacto-ovo vegetarians, 1 vegan, 1 lacto-vegetarian, and 1 ovo-vegetarian. All patients had constant and stable vegetarian behaviors during ESRD while undergoing renal replacement therapy. The patients followed vegetarian diets for religious reasons, rather than for body weight control or for treatment of illness. The median and average durations of vegetarian diet were 300 months and 286.29 ± 128.41 months, respectively.

Clinical characteristics were compared between the two groups (Tables 1 and 2). The vegetarian group had a lower body weight and body mass index (*p* < 0.05). The serum BUN, serum creatinine, uric acid, serum phosphate, serum calcium × phosphate products, 25(OH) D, serum albumin, and nPCR levels were significantly lower in the vegetarian group than in the non-vegetarian group (*p* < 0.05). In contrast, the vegetarian group had significantly higher serum triglyceride levels than the non-vegetarian group (*p* < 0.05). There were no significant differences in age, duration of dialysis, systolic arterial pressure, mean arterial pressure, diastolic arterial pressure, hematocrit, hemoglobin, total cholesterol, HDL, LDL, alkaline phosphatase, serum calcium levels, iPTH, Hs-CRP, homocysteine, or serum vitamin B12 level between the two groups.

**Table 1:** Demographic differences between the vegetarian and non-vegetarian groups.

Parameter	Vegetarians (n = 21)	Non-vegetarians (n = 42)	*p* value
Sex (male/female)	4/17	8/34	1.000
Age (years)	56.24 ± 13.71	56.29 ± 11.67	0.989
Duration of dialysis (months)	112.20 ± 80.37	131.77 ± 64.81	0.304
Body weight (kg)*	50.26 ± 7.18	55.88 ± 9.18	0.018
Body mass index (kg/m^2^)*	20.39 ± 2.14	22.46 ± 3.38	0.013
Duration of vegetarian (months)	286.29 ± 128.41	–	–
Underlying disease			
Chronic interstitial nephritis	13 (61.90%)	19 (45.24%)	0.212
Chronic glomerular nephropathy	5 (23.81%)	17 (40.48%)	0.191
IgA nephropathy	1 (4.76%)	4 (9.52%)	0.657
Autosomal dominant polycystic kidney disease	1 (4.76%)	1 (2.38%)	1.000
Lupus nephritis	1 (4.76%)	1 (2.38%)	1.000
Comorbid conditions			
Smoking	0	1 (2.38%)	1.000
Previous MI episode	0	4 (9.52%)	0.292

Values are expressed as mean ± standard deviation

*p < 0.05

**p < 0.01

**Table 2:** Differences in biochemical and nutritional markers between the vegetarian and non-vegetarian groups.

Parameter	Vegetarians (n = 21)	Non-vegetarians (n = 42)	*p* value
Hemoglobin	10.53 ± 1.32	10.54 ± 1.17	0.994
Hematocrit	32.00 ± 3.96	32.39 ± 3.72	0.706
Blood urea nitrogen (mg/dL)**	51.66 ± 13.78	67.58 ± 16.43	<0.001
Serum creatinine (mg/dL)**	8.55 ± 1.84	10.35 ± 1.72	<0.001
Triglyceride (mg/dL)**	205.24± 123.47	141.76 ± 107.95	0.003
Total cholesterol (mg/dL)	186.86 ± 44.20	196.05 ± 34.19	0.366
High-density lipoprotein cholesterol (mg/dL)	44.71 ± 12.94	48.12 ± 17.65	0.436
Low-density lipoprotein cholesterol (mg/dL)	87.52 ± 26.77	93.19 ± 26.86	0.432
Uric acid (mg/dL)**	5.74 ± 1.58	7.10 ± 1.45	0.001
Albumin (g/dL)**	3.77 ± 0.35	4.02 ± 0.35	0.009
Calcium (mg/dL)	9.58 ± 0.90	9.77 ± 0.76	0.382
Serum phosphate (mg/dL)**	3.96 ± 1.27	5.36 ± 1.50	<0.001
Calcium x Phosphate (mg^2^/dL^2^)**	37.74 ± 12.13	52.44 ± 15.25	<0.001
Alkaline phosphatase (U/L)	119.19 ± 70.57	119.38 ± 93.46	0.884
Intact parathyroid hormone (pg/mL)	328.92 ± 362.96	600.78 ± 584.27	0.139
High-sensitivity C-reactive protein (mg/dL)	0.67 ± 0.98	0.66 ± 1.12	0.354
Normalized protein catabolic rate (g/day/kg)*	1.02 ± 0.27	1.24 ± 0.36	0.011
Homocysteine level (umol/L)	21.62 ± 7.62	24.97 ± 9.71	0.088
Vitamin B12 level (pg/mL)	901.70± 665.00	994.80 ± 568.97	0.243
25 (OH) Vitamin D level (ng/mL)**	14.43 ± 8.00	23.78 ± 9.68	<0.001
25 (OH) Vitamin D level ≦10 (ng/mL) **	7/21	2/42	0.005

Values are are expressed as mean ± standard deviation.

* *p* < 0.05

** *p* < 0.01

As shown in Table 3, dietary compositions were compared between different groups using 24-hour dietary record analysis. The vegetarian group consumed less protein and fat than the non-vegetarian group (*p* < 0.05). No significant differences were found in total energy gain between these two groups. Values for baPWV were significantly higher in the vegetarian group compared with the non-vegetarian group (1926.95 ± 456.45 cm/sec vs. 1684.82 ± 309.55 cm/sec, respectively; *p* < 0.05) (Table 4). Independent factors associated with baPWV were further evaluated using multiple linear regression analysis (Table 5). Vegetarian diet remained an independent risk factor for elevated baPWV, even after adjusting for age, pre-dialysis systolic arterial blood pressure, duration of dialysis, and serum albumin.

**Table 3:** Comparison of dietary composition between vegetarians and non-vegetarians.

	Vegetarians (n = 21)	Non-vegetarians (n = 42)	*p* value
Energy (Cal)	1439.66 ± 404.64	1669.47 ± 512.02	0.078
Protein (g)*	47.29 ± 15.42	60.57 ± 21.88	0.016
Carbohydrate (g)	197.98 ± 61.75	211.83 ± 75.87	0.472
Fat (g)*	48.71 ± 16.55	57.36 ± 19.39	0.012

Values are expressed as mean ± standard deviation

* *p* < 0.05

** *p* < 0.01

**Table 4:** Comparison of index of artery stiffness between vegetarian and non-vegetarian groups.

Parameter	Vegetarians (n = 21)	Non-vegetarians (n = 42)	*p* value
Systolic arterial blood pressure (mmHg)	139.71± 18.52	141.79 ± 24.88	0.737
Mean arterial blood pressure (mmHg)	107.67 ± 13.31	108.28 ± 18.14	0.890
Diastolic arterial blood pressure (mmHg)	82.14 ± 9.98	80.79 ± 12.77	0.878
Brachial-ankle pulse wave velocity (cm/sec)*	1926.95± 456.45	1684.82 ± 309.55	0.036
Ankle-brachial index	1.16 ± 0.10	1.16 ± 0.11	0.956

Values are expressed as mean ± standard deviation

* *p* < 0.05

** *p* < 0.01

**Table 5:** Output from multiple stepwise regression analyses between multiple factors and brachial-ankle pulse wave velocity in 63 dialysis patients.

Parameter	β	95% CI	*p* value
Vegetarian diet	0.256	49.163–358.986	0.011
Age (years)	0.499	9.400–21.413	<0.001
Pre-dialysis systolic blood pressure (mmHg)	0.337	2.476–8.713	0.001
Duration of dialysis (months)	-0.093	-1.563–0.555	0.342
Albumin	-0.154	-366.478–44.780	0.123

The regressions coefficients and 95% coefficient interval value are indicated. All models are after adjustment for all above covariates including diet, age, and pre-dialysis systolic blood pressure, duration of dialysis and serum albumin.

## Discussion

The major findings of this study were: (1) patients who followed vegetarian diets had lower serum BUN, creatinine, phosphate, serum calcium × phosphate products, uric acid, vitamin D, albumin, and nPCR levels; and (2) patients who followed vegetarian diets had increased vascular stiffness as assessed by baPWV compared with non-vegetarians. Kuang et al. [17] previously showed that baPWV had an independent correlation with age and serum albumin level among peritoneal patients. Additionally, Kim et al. [18] found that age, pre-dialysis systolic blood pressure, and diabetes were independent variables of baPWV in ESRD patients. After adjusting for these known factors, we still found that vegetarian diet was a novel independent risk factor for vascular stiffness in chronic dialysis patients.

In Taiwanese society, vegetarianism commonly originates from personal or family religious reasons (i.e., Buddhism and Taoism), and has been practiced for centuries. As part of Taoist and Buddhist practice, followers have incorporated lifestyle rituals including vegetarianism, good moral conduct, and the use of appropriate incantations, amulets, and charms. In this study, all patients had chosen a vegetarian diet for religion reasons and not as a treatment for a previous illness. Furthermore, patients were enrolled in the vegetarian diet group only if they had consistent and stable diet behavior. The average duration of vegetarian diet was 286.29 ± 128.41 months. Therefore, the vegetarian group in this study can be considered to represent the true effects of a vegetarian diet on biochemical and vascular functions, unbiased by personal disease.

In the present study, the vegetarian group had significantly lower serum BUN, creatinine, phosphate, calcium × phosphate, uric acid, albumin, and nPCR levels compared with the non-vegetarian group. The nPCR, known as the protein equivalent of nitrogen appearance, is an indicator of protein catabolism and appropriately reflects dietary protein intake in a steady condition. Albumin can serve as indicator of visceral protein storage. These markers showed that vegetarian diets include lower protein intake than non-vegetarian diets. All data generated in this study, including 24-hour dietary record analyses, support the hypothesis that usual Taiwanese vegetarian diets have lower protein content and decreased nitrogen load, resulting in lower BUN, phosphate, and serum uric acid levels. These findings were compatible with those from a previous study [19].

In the present study, the vegetarian group showed no significant differences in serum homocysteine or vitamin B12 levels compared with the non-vegetarian group. Homocysteine is a byproduct of methionine metabolism, a sulfhydryl-containing essential amino acid that is observed at the junction of two metabolic pathways: transsulfuration and re-methylation. Although it is highly possible that patients who are on chronic dialysis and consume a vegetarian diet may have lower serum vitamin B12 levels, which may induce higher serum homocysteine levels, this was not observed in the present study. In Taiwan, water-soluble vitamins including B12 have generally been provided for chronic dialysis patients in recent decades. As a result, serum vitamin B12 and homocysteine levels were not observed to be significantly different between different diets in this study.

We demonstrated significantly lower serum 25(OH) D levels in patients who consumed vegetarian diets. This finding was compatible with results from a previous study in Finland, in which strict Western vegetarian diets were found to be at risk for vitamin D deficiency in the winter, primarily due to low dietary vitamin D3 intake and despite normal sunlight exposure in the summer [20]. Humans obtain vitamin D through exposure to sunlight, diet, and dietary supplements. Foods that provide the highest quantities of natural vitamin D3 per gram all come from animal sources, primarily fish oil. However, the dietary resources (plant, yeast, and fungi) available to vegetarians only contain Vitamin D2. Vitamin D3 is more potent in raising and maintaining serum 25(OH) D concentrations and leads to 2- to 3-fold greater storage of vitamin D than does equimolar vitamin D2 [21]. In our study, vegetarian diets contained lower protein intake; this was confirmed by 24-hour dietary records and normalized protein catabolic rate (Tables 2 and 3). Vegetarian diets appear to result in higher risk of 25(OH) D deficiencies due to insufficient protein intake and vitamin D3 deficiencies.

The influence of vegetarian diet on vascular stiffness remains a major issue, especially for high-risk groups such as chronic dialysis patients. The primary endpoint of baPWV was chosen in this study as a surrogate for CVD risk. However, presence of severe PAOD decreases baPWV due to decreased blood flow and internal pressure. Arrhythmias, such as atrial fibrillation and atrioventricular block, also interfere with the results of baPWV analysis. In the present study, patients with atrial fibrillation, high-degree atrioventricular block, and PAOD had waveform images that were inadequate for the calculation of baPWV. Hence, all confounding factors of baPWV were excluded. BaPWV measurement in this study was a valid and acceptable representation of aortic PWV measured using conventional methods. Vascular stiffness in vegetarians, assessed by baPWV, was worse than that in non-vegetarians. Consuming a vegetarian diet was an independent risk factor for vascular stiffness, even after adjusting for other known factors of arterial stiffness (Table 5). Vegetarian diet resulting in 25(OH) D deficiencies may be a possible etiology. The vitamin D hormone is essential for the preservation of serum calcium and phosphate levels, but may also be a regulator of endothelial nitric oxide synthase and arterial stiffness [22]. As many previous studies have revealed, lower serum 25(OH) D concentrations are associated with higher arterial calcification score, more LV hypertrophy and dilatation, and increased risk of cardiovascular events in patients on chronic dialysis [23–25]. Furthermore, a vegetarian diet may also lead to low dietary protein intake. A low protein diet has been confirmed to enhance vascular calcification in uremic rats with hyperphosphatemia [26]. The combination of these factors may limit the beneficial cardiovascular effects of a vegetarian diet and even result in adverse effects.

Data seen in Table 3 show that fat intake is significantly higher in non-vegetarians. Such high intake of fat is not conducive from a cardiac point of view. Although it would seem intuitive to extrapolate recommendations for low dietary fat in the general population, virtually no convincing data exist to suggest that restricting dietary fat has any advantage in dialysis groups. A surprising observation, which is not well known, is that in most epidemiologic studies lower body fat is associated with greater mortality in dialysis patients [27]. Previous study showed that tissue levels of long-chain n-3 fatty acids are depressed in vegetarians [28]. n-3 fatty acids have a beneficial effect on the vascular system, reducing pulse wave velocity and modulating inflammation [29]. For example, vegetarians have much lower plasma concentrations of eicosapentaenoic acid and docosahexaenoic acid, both of which are strongly related to human health. Vegetarians do not eat fish and thus consume virtually no eicosapentaenoic acid and docosahexaenoic acid. Besides, conversion of the plant-derived n-3 fatty acid α-linolenic acid to eicosapentaenoic acid and docosahexaenoic acid is very low. Despite similar lipid profiles in this study (Table 2), vegetarian diets with lower fat intake may have offset the beneficial cardiovascular effects of vegetarianism. Furthermore, it is possible, although not yet proven, that properly administered dietary fat could have additional benefits on the vessel system [30, 31].

There were several limitations to the present study that must be considered. Firstly, causal relationships could not be determined owing to the cross-sectional study design. Secondly, the study sample size was relatively small; hence, the study may not have been adequately powered to detect other potential risk factors. Thirdly, patients with malignancy, diabetes mellitus, or active inflammation have been excluded; thus, the results cannot be applied to these populations. Despite these limitations, this was the first study investigating the effects of Taiwanese vegetarianism on nutritional and cardiovascular status in chronic dialysis patients.

In conclusion, enforcing dialysis-related dietary restrictions in vegetarian Taiwanese patients with ESRD may contribute to subclinical protein malnutrition and vitamin D deficiency, offsetting the beneficial cardiovascular effects of vegetarianism. A prospective randomized controlled study will be needed to evaluate whether correction of vitamin D deficiency and protein supplement will improve cardiovascular outcomes in patients with ESRD who are vegetarians.

## Supporting Information

S1 File Clinical data of the participants. Underlying participant-level data are supplied in a supporting information file.
(XLSX)
